# Supplementary material for: Antisite occupation induced single anionic redox chemistry and structural stabilization of layered sodium chromium sulfide
Source: Nat Commun. 2017 Sep 18;8:566. doi: 10.1038/s41467-017-00677-3 (PMC5603526; doi:10.1038/s41467-017-00677-3)
Supplement: Supplementary file 1 — Supplementary Information [file 41467_2017_677_MOESM1_ESM.pdf]

Description of Supplementary Files

File Name: Supplementary Information
Description: Supplementary Figures, Supplementary Tables, Supplementary Notes and Supplementary References.

File Name: Peer Review File

## Supplementary Figures

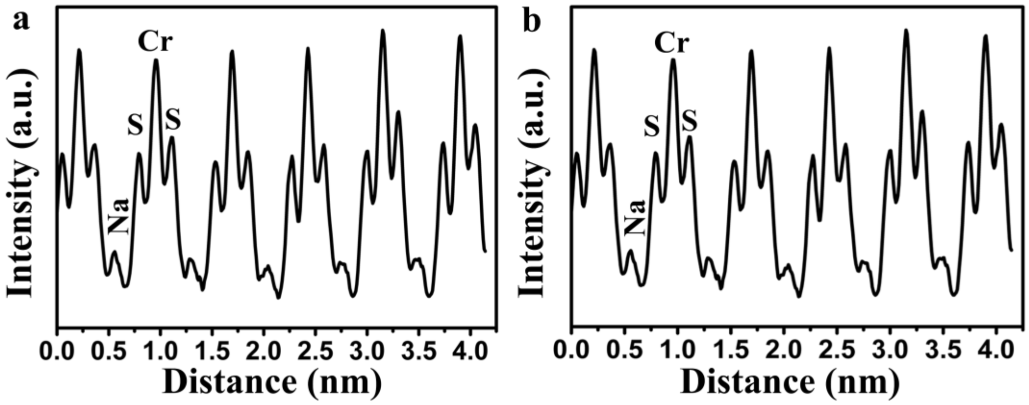


**Supplementary Figure 1 | Atomic structure of pristine $NaCrS_2$.** (a) Average image intensity profiles of the regions designated by (a) line 1 (surface) and (b) line 2 (bulk) in HAADF-STEM of pristine $NaCrS_2$ as shown in figure 1b.

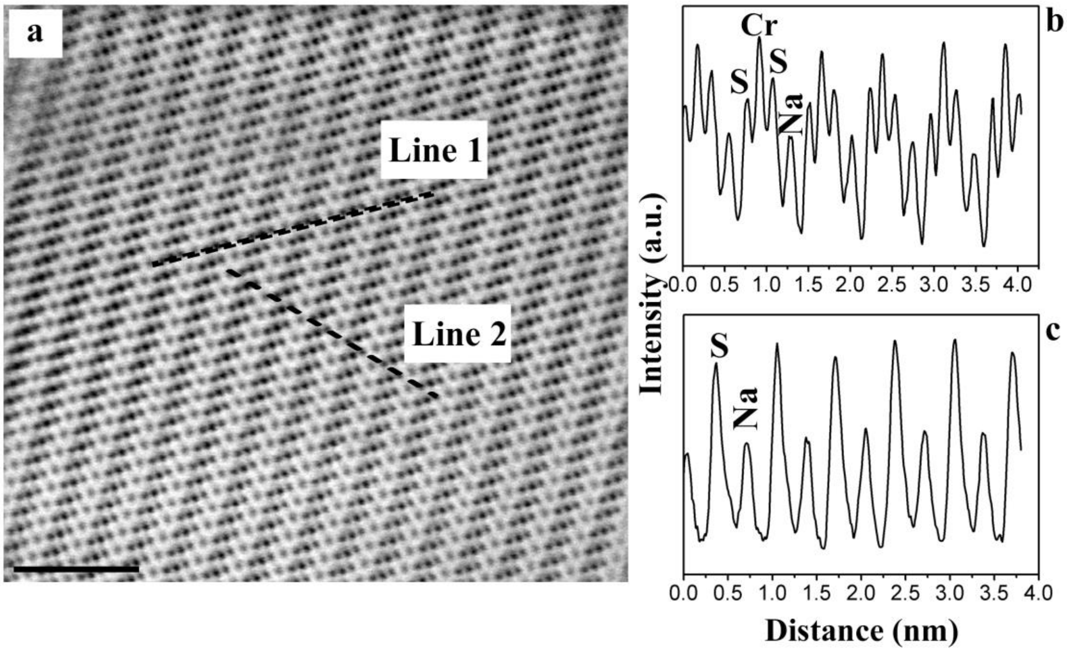


**Supplementary Figure 2 | Atomic structure of pristine $NaCrS_2$.** (a) The full atomic resolution ABF-STEM image for the pristine particle, scale bar 2 nm (b) and (c) are the average image intensity profiles of the regions designated by line 1 and line 2, respectively. Image contrast of the dark dots is inverted and displayed as peaks.

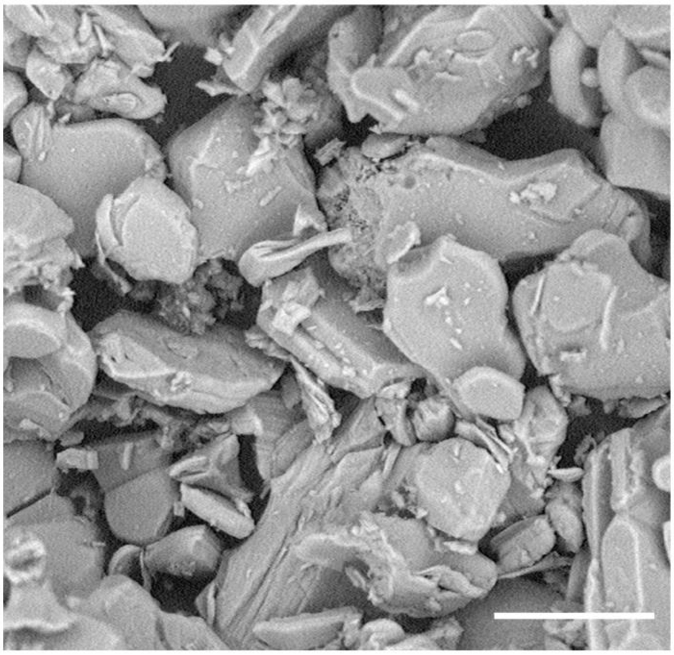

**Supplementary Figure 3 | Morphology of pristine $NaCrS_2$.** SEM image of as-prepared $NaCrS_2$ powder, scale bar: 5 μm.

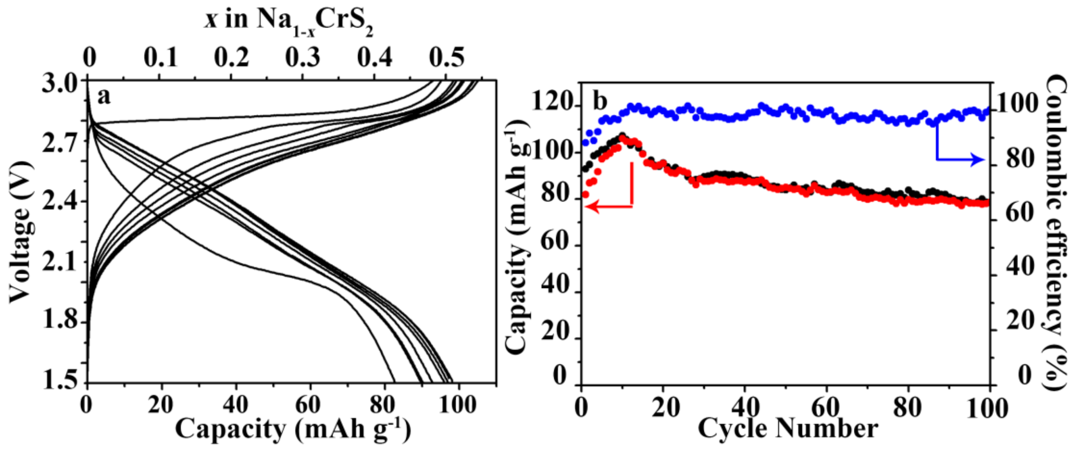


**Supplementary Figure 4 | Chare/discharge performance at 0.1 C.** (a) Galvanostatic charge/discharge profiles, (b) cyclic performance and Coulombic efficiency of $NaCrS_2$ electrode under current density of 0.1 C.

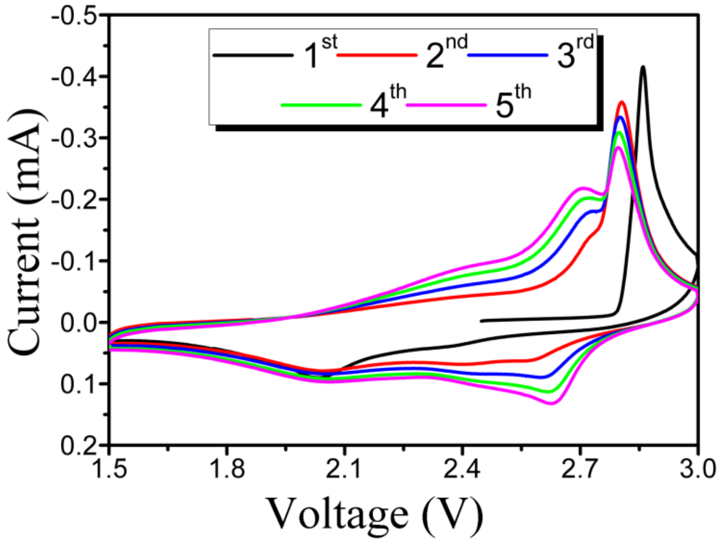


**Supplementary Figure 5 | Cyclic voltammogram profile.** (a) First five cyclic voltammograms for the $NaCrS_2$ electrode cycled between 1.5 to 3.0 V at a scan rate of 0.1 mV $s^{-1}$.

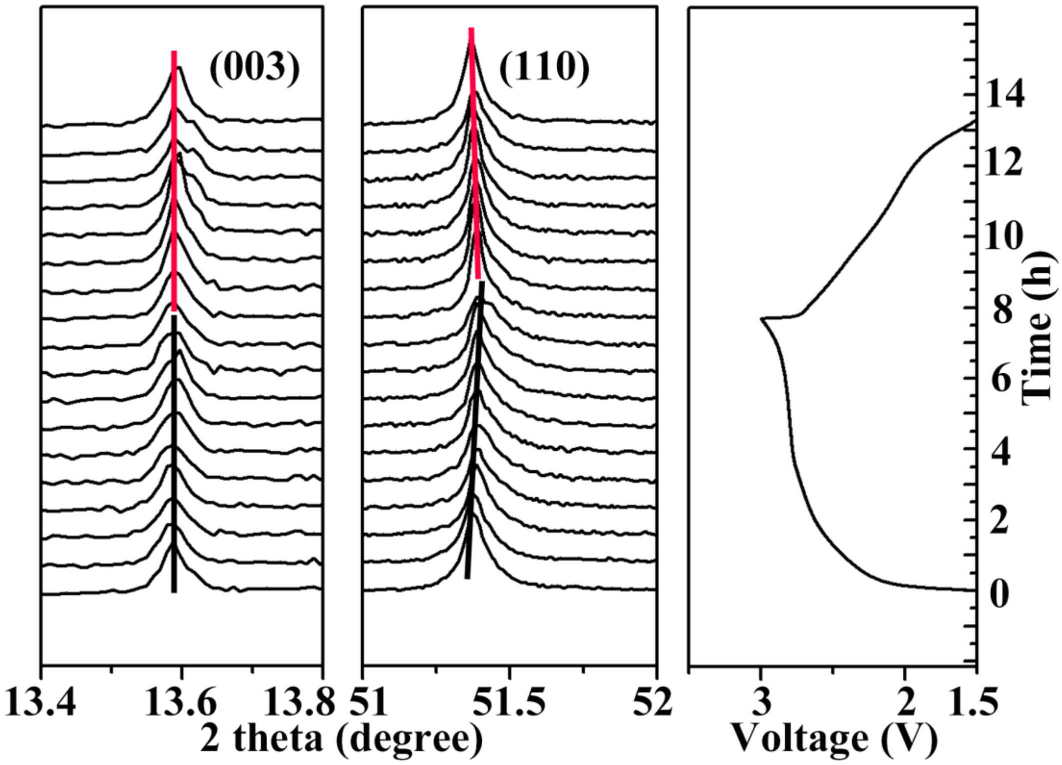


**Supplementary Figure 6 | Na-driven structural evolution during 3rd cycle.** *In situ* XRD patterns collected during the third charge-discharge cycle for $NaCrS_2$, corresponding time vs. voltage profile is shown on the right. (The $2\theta$ is converted to the corresponding angle for $\lambda$=1.54 Å (Cu-Kα) from the real wavelength $\lambda$=0.7747 Å used for synchrotron XRD experiments).

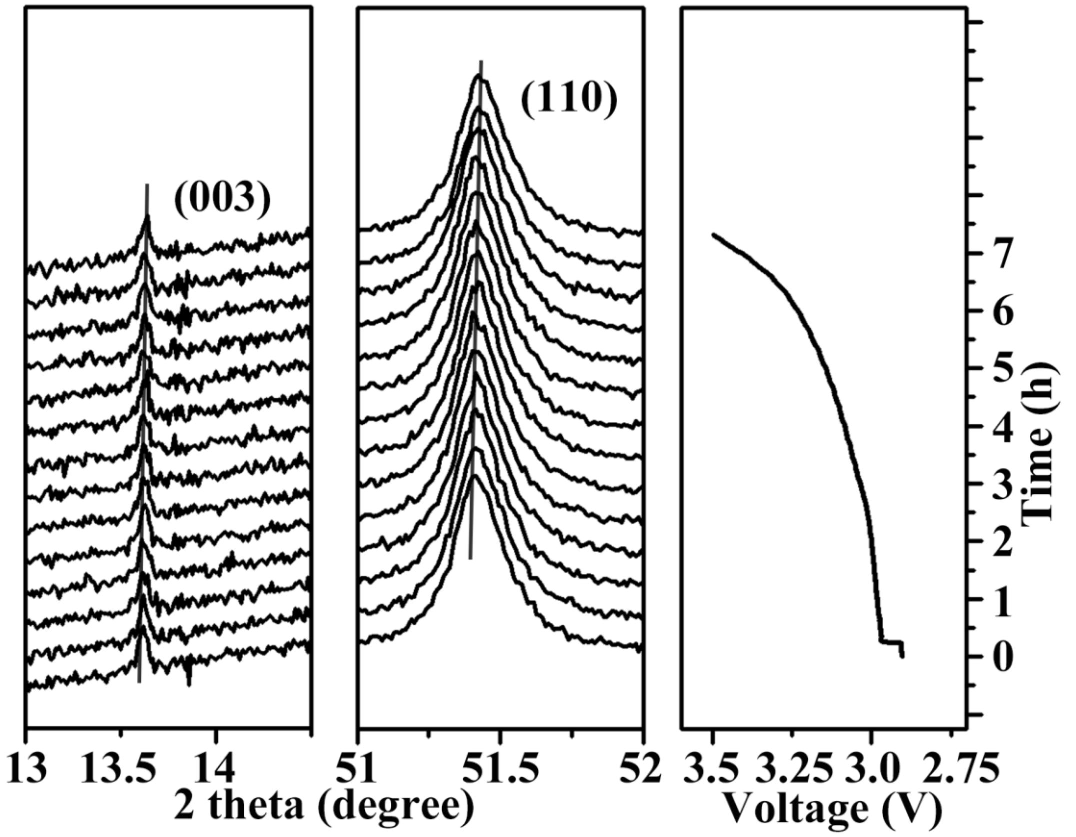


**Supplementary Figure 7 | Li-driven structural evolution for $NaCrS_2$/Li cell.** *In situ* XRD patterns collected during the first charging process for $NaCrS_2$/Li cell, corresponding time vs. voltage profile is shown on the right. (The $2\theta$ is converted to the corresponding angle for $\lambda$=1.54 Å (Cu-K$\alpha$) from the real wavelength $\lambda$=0.7747 Å used for synchrotron XRD experiments).

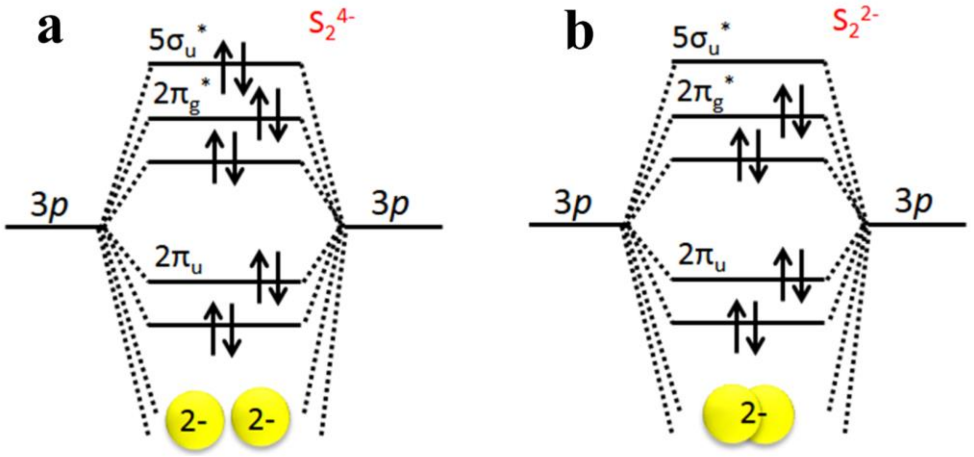


**Supplementary Figure 8 | Schematic of the '$S_2$' molecular orbital diagram.** The '$S_2$' molecular orbital diagrams of (a) $(S_2)^{4-}$ and (b) $(S_2)^{2-}$.

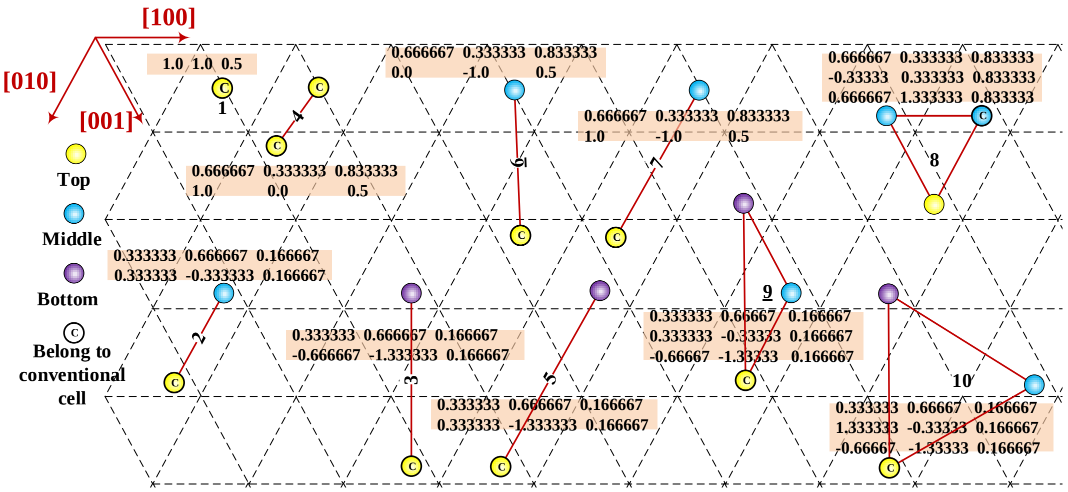


**Supplementary Figure 9 | Clusters with effective cluster interaction (ECI) in cluster expansion in $NaCrS_2$.** The point cluster, pair cluster and triplet cluster are denoted by single point, straight line and triangle, respectively.

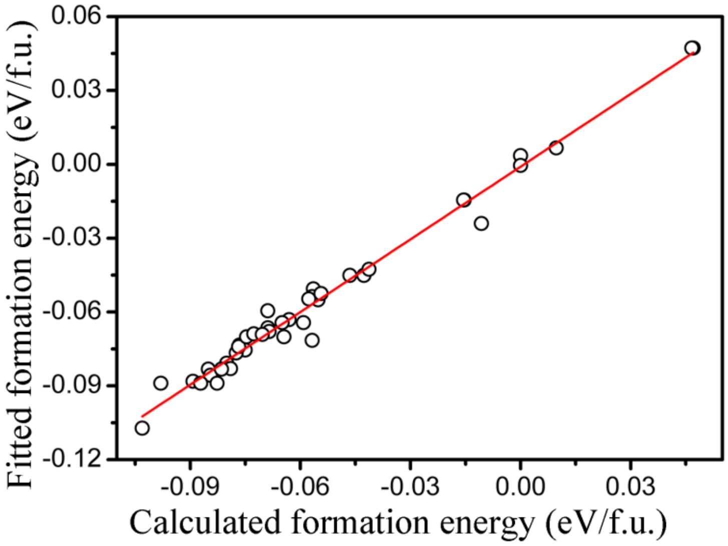


**Supplementary Figure 10 | Fitting residual of formation energies**. This is the difference between the first-principles energies and the energies predicted from the cluster expansion. The Cross-validation score is 0.00931249.

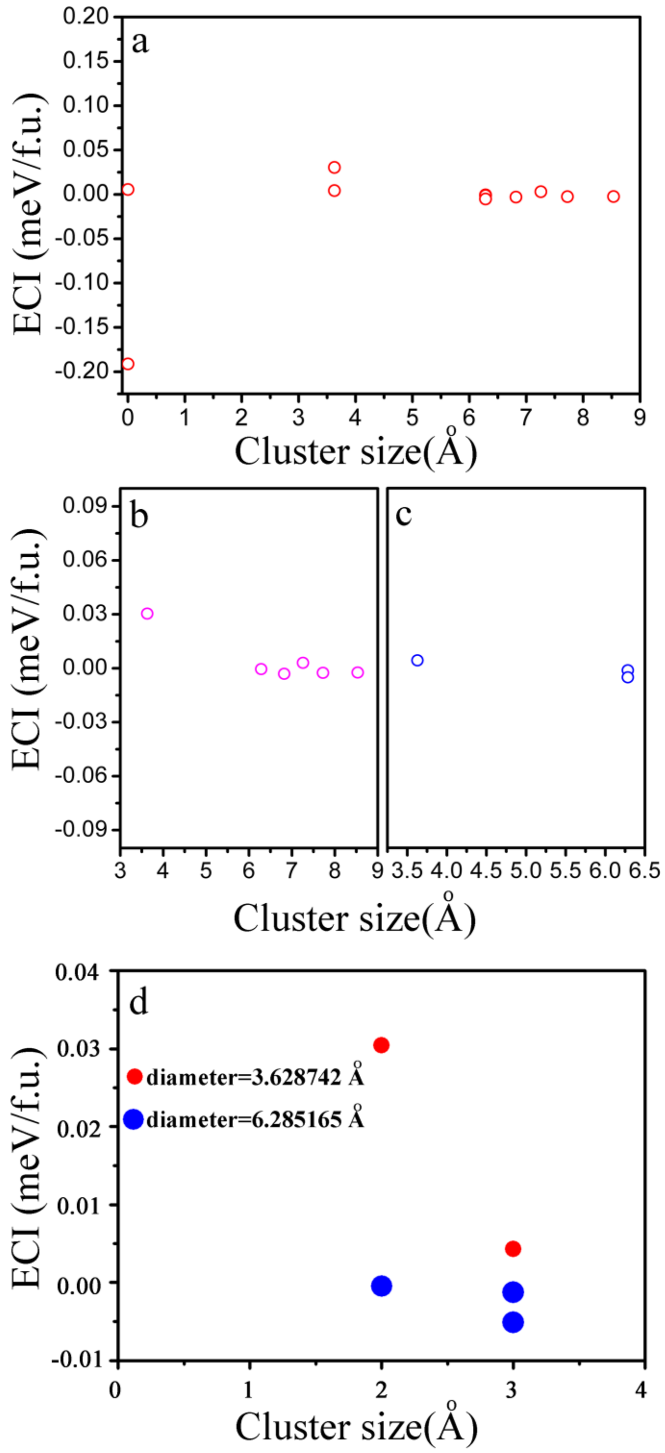


**Supplementary Figure 11 | ECI values for the cluster expansion.** ECI value for the cluster expansion, which decays as a function of diameter of the corresponding cluster and also a function of the cluster size. Cluster diameter means the maximum distance between any two sites in the cluster. Cluster size means the number of sites in the cluster (i.e. null, point, pair, triplet). (a) ECI values for all clusters. (b) The ECI values

for pair clusters and (c) for triplet clusters. (d) ECI values for the same-diameter clusters. With the same cluster size (pair cluster or triplet cluster), the ECI value decreases when the cluster length increases. With the same cluster diameter (*i.e*. 6.285165 Å, 3.628742 Å), the ECI value decreases when the cluster size increases.

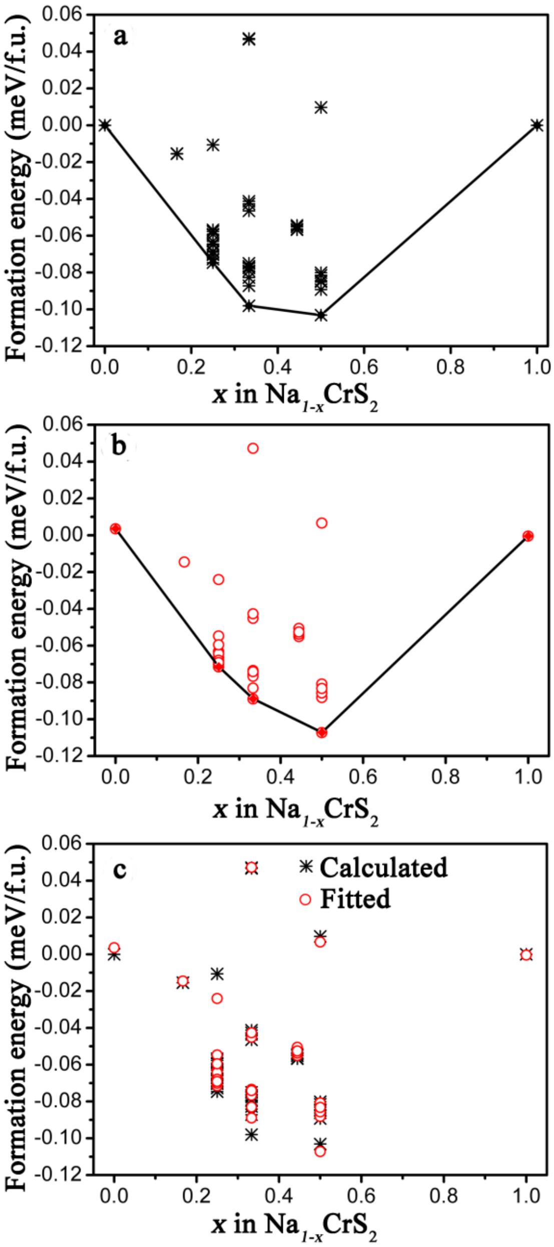


**Supplementary Figure 12 | Formation energies of ground states.** Ground state refers to a configuration with lowest formation energies, which can be considered as most stable configuration (Ground state: A configuration with lowest formation energies, which can be considered as the most stable configuration. All of the ground states at the different concentrations are located at convex hull.) (a) Calculated formation energies per formula unit as a function of Na content in $Na_{1-x}CrS_2$. (b) Fitted formation energies per formula unit as a function of Na content in $Na_{1-x}CrS_2$. (c) Comparison between calculated and fitted formation energies per formula unit as a function of Na content in $Na_{1-x}CrS_2$.

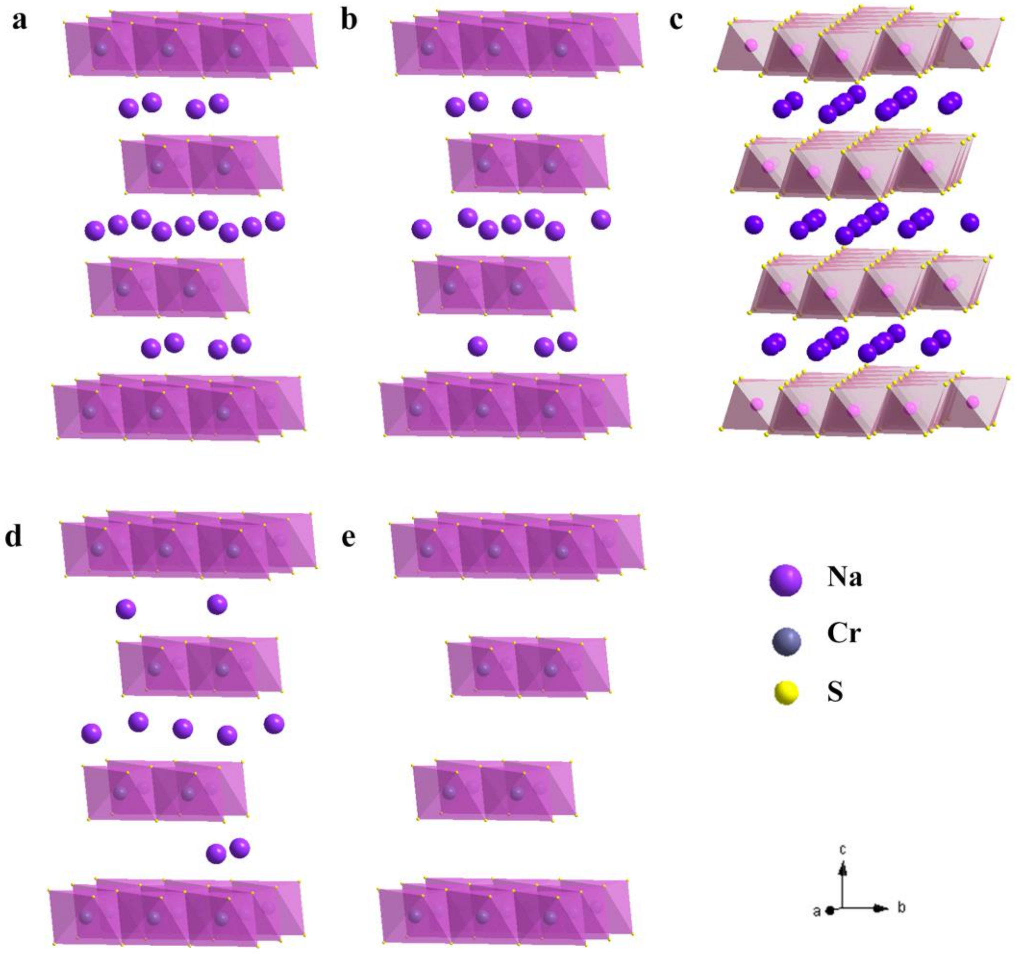


**Supplementary Figure 13 | Ground-state crystal structures.** Ground-state structures of Na-vacancy concentrations of (a) 0, (b) 1/4, (c) 1/3, (d) 1/2, and (e) 1.0.

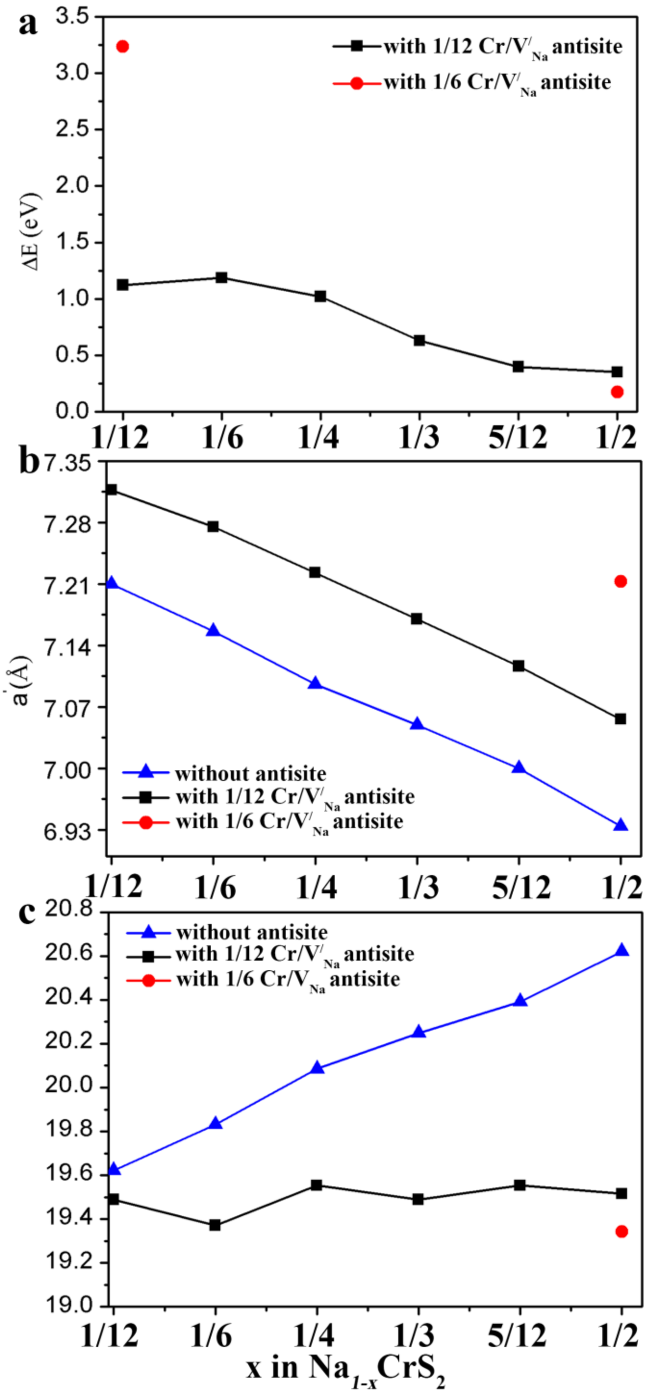


**Supplementary Figure 14 | $Na_{1-x}CrS_2$ with $Cr/V^{/}_{Na}$ antisite.** (a) Calculated total energy differences Δ*E* between configurations (black square: with 1/12 $Cr/V^{/}_{Na}$ antisite, red circle: with 1/6 $Cr/V^{/}_{Na}$ antisite), (b) calculated lattice parameters *c* and (c) calculated lattice parameters *a′* (black square: without antisite, red circle with 1/12 Cr/ $V^{/}_{Na}$ antisite, blue triangle: with 1/6 Cr/ $V^{/}_{Na}$ antisites) of 2×2×1 $Na_{1-x}CrS_2$ (*x*=0, 1/12, 1/6, 1/4, 1/3, 5/12, 1/2) conventional cells with and without $Cr/V^{/}_{Na}$ antisites.

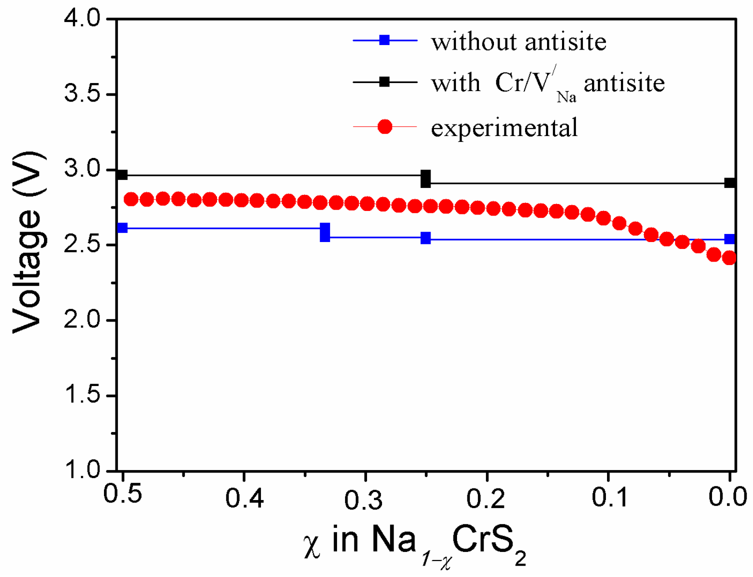


**Supplementary Figure 15 | Calculated average potential.** Galvanostatic intermittent titration technique (GITT) profiles (Red line) and calculated voltage (Black line and blue line) of $Na_{1-x}CrS_2$ with and without antisite.

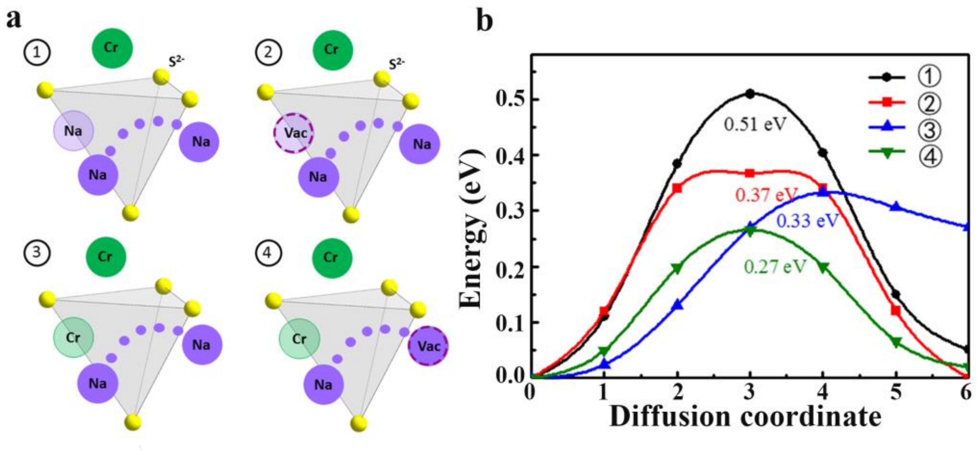


**Supplementary Figure 16 | Na diffusion by hopping from one octahedral site to another octahedral site via an intermediate tetrahedral site (*o*-*t*-*o* diffusion).** (a) Possible environments for an *o*-*t*-*o* Na hopping in ① $Na_{0.75}CrS_2$ without $Cr/V^{/}_{Na}$ antisite; ② $Na_{0.667}CrS_2$ without $Cr/V^{/}_{Na}$ antisite (derived due to one $Na^+$ deintercalation from $Na_9Cr_{12}S_{24}$ supercell corresponding to $Na_{0.75}CrS_2$ in ①); ③ $Na_{0.5}CrS_2$ with 1/6 $Cr/V^{/}_{Na}$ antisite; ④ $Na_{0.417}CrS_2$ with 1/6 $Cr/V^{/}_{Na}$ antisite (derived due to one $Na^+$ deintercalation from $Na_6Cr_{12}S_{24}$ supercell corresponding to $Na_{0.5}CrS_2$ in ③). (b) Calculated Na hopping barriers through *o*-*t*-*o* diffusion pathways in ①-④.

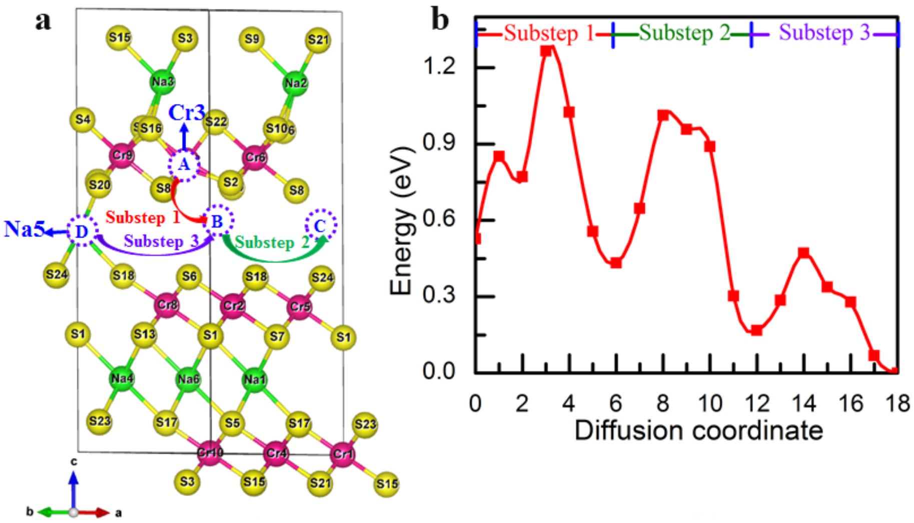


**Supplementary Figure 17 | Cr migration in $Na_{0.5}CrS_2$.** (a) Possible diffusion path and (b) corresponding energy profile. Substeps 1 and 2 denote the migration process of Cr (Cr3) from the lattice site A to the vacancy at site B and then to the vacancy at site C, respectively. Substep 3 is the migration process of Na (Na5) from the lattice site D to the vacancy at site B.

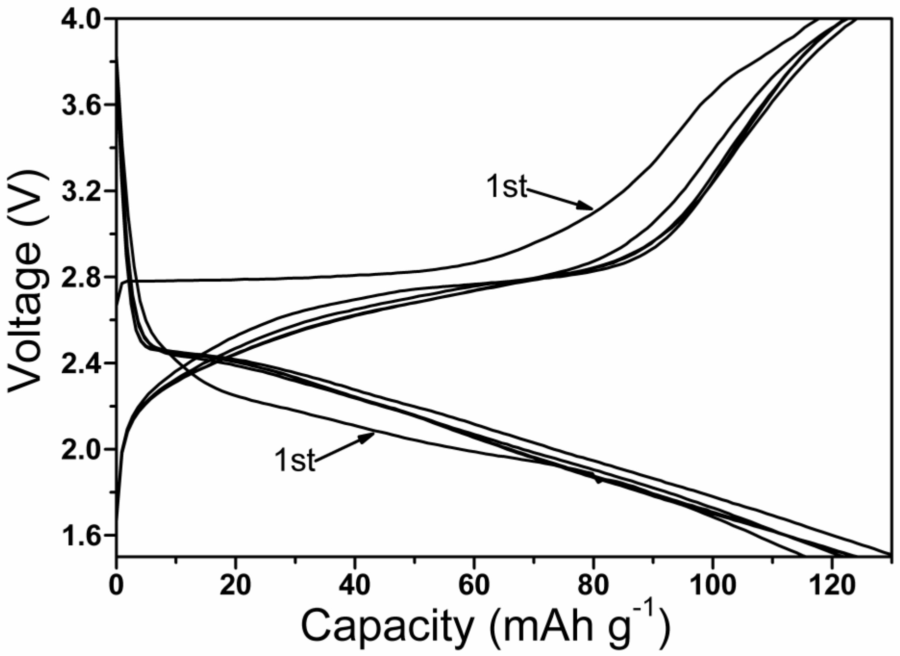


**Supplementary Figure 18 | Galvanostatic charge/discharge curves $NaCrS_2$ in a wide voltage range.** Charge/discharge curves of $NaCrS_2$ electrode cycled between 1.5-4.0 V at a current density of 0.1 C.

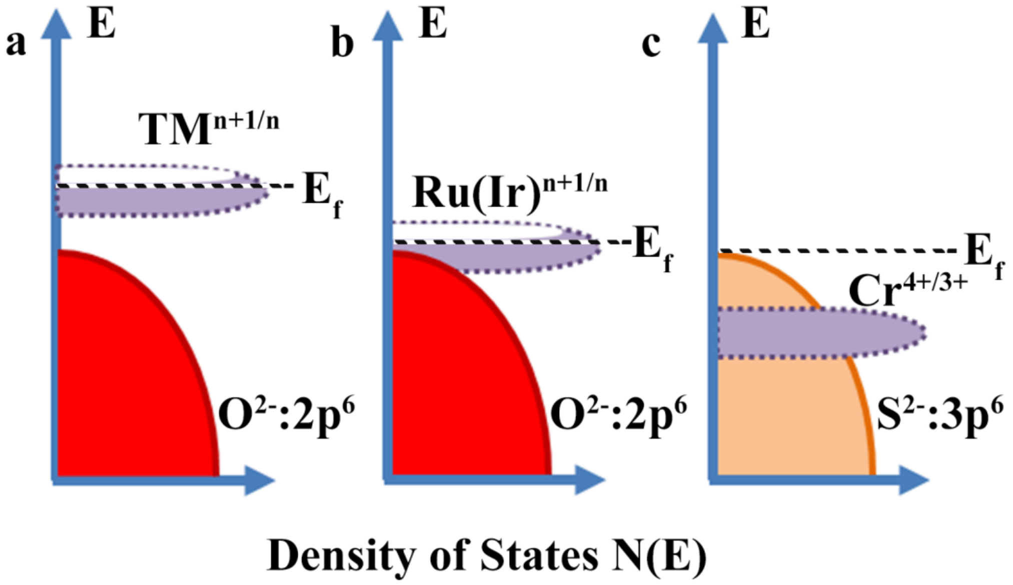


**Supplementary Figure 19 | Schematic representation of DOS.** Schematic representation of different redox couples with possible positions relative to the top of the anion p bands. (a) Positions of common $TM^n/TM^{n+1}$ redox couples; (b) Positions of $Ru(Ir)^n/Ru(Ir)^{n+1}$ and $O^{2-}/O_2^{2-}$ redox couples; and (c) Positions of $S^{2-}/S_2^{2-}$ redox couple.

# Supplementary Tables

**Supplementary Table 1**. Structural parameters and atomic positions of as-prepared $NaCrS_2$ deducted from Rietveld Refinement of Synchrotron Data.

| Atom | Wyckoff | Occupancy | *x*/a | *y*/b | *z*/c | $U_{iso}$ (Å$^2$) |
|---|---|---|---|---|---|---|
| Na | 3b | 1 | 0 | 0 | 0.5 | 0.01701 |
| Cr | 3a | 1 | 0 | 0 | 0 | 0.00578 |
| S | 6c | 1 | 0 | 0 | 0.267076 | 0.01043 |
| *R-3m*: | *a* = *b* = 3.5270(4) Å | | | *c* = 19.3506(1) Å | | |
| | $R_p$ = 2.9% | | $R_{wp}$ = 3.9% | GOF($\chi^2$) = 4.67 | | |

**Supplementary Table 2**. Cr K-edge EXAFS structure parameters of $NaCrS_2$ at different charge states.

| Samples | Path | $r$/Å | $\sigma^2$ /$10^{-3}$Å$^2$ | $\Delta E$/eV | $R$ |
|---|---|---|---|---|---|
| $NaCrS_2$ | Cr-S(6) | 2.42(1) ± 0.006 | 4.79 ± 0.83 | 2.15 ± 0.78 | 0.007 |
| | Cr-Cr | 3.57(9) ± 0.009 | 6.14 ± 1.08 | | |
| $NaCrS_2$ charge | Cr-S1(4) | 2.41(5) ± 0.015 | 2.72 ± 1.01 | 0.42 ± 1.86 | 0.008 |
| | Cr-S2(2) | 2.33(8) ± 0.027 | 13.84 ± 16.91 | | |
| | Cr-Cr | 3.55(6) ± 0.019 | 7.96 ± 2.18 | | |

$r$: bond length; $\sigma^2$: Debye-Waller factor (disorder); $\Delta E$: inner shell potential shift; $R$: R-factor.

**Supplementary Table 3**. Calculated and fitted formation energies of $Na_{1-x}CrS_2$[a] ($0 \leq x \leq 1$), and Na-vacancy coordinates of ground states.

| Concentration of Vacancy ($x$) | Calculated formation energy (eV/f.u.) | Fitted formation energy (eV/f.u.) | Fractional coordinates of vacancy[a] |
|---|---|---|---|
| 0.000000 | 0.000000 | 0.003537 | |
| 0.250000 | -0.074664 | -0.070084 | (0.5, 0, 0.5) (0.33, 0.17, 0.83) (0.67, 0.33, 0.17) |
| 0.333333 | -0.098012 | -0.088927 | (0, 0, 0.5) (0.67, 0.33, 0.17) (0.33, 0.67, 0.17) |
| 0.500000 | -0.103099 | -0.107172 | (0.33, 0.17, 0.83) (0.17, 0.33, 0.17) (0.5, 0, 0.5) (0.67, 0.33, 0.17) (0, 0.5, 0.5) (0.83, 0.67, 0.83) |
| 1.000000 | 0.000000 | -0.000444 | |

[a]Coordinates of the Na-vacancy are given as fractional coordinates of the 2×2×1 conventional cell for the concentrations of 0.25 and 0.5 and √3×√3×1 one for the case of 0.333333.

**Supplementary Table 4**. Lattice parameters and the corresponding total energies for $Na_{1-x}CrS_2$ ($0 \le x < 1$) with and without $Cr/V^{/}_{Na}$ antisites. The difference between two $Na_{0.5}CrS_2$ configurations with $Cr/V^{/}_{Na}$ antisites (See Supplementary Fig. 13d and Fig. 17a for 2×2×1 $Na_{0.5}CrS_2$ conventional cells) lies in that three Na layers for the former have the same $Na^+$ numbers, whereas the $Na^+$ number of the middle Na layer for the latter is less than those of the two-sided ones so as to ensure the enough Na vacancies for Cr migration. As for $Na_{0.5}CrS_2$ with 1/12 $Cr/V^{/}_{Na}$ antisite derived from Supplementary Fig. 17a, three possible configurations with the energy difference of ~0.036eV/f.u. are listed. Note that due to the usage of 2×2×1 conventional cell, $a'$ is equal to $2a$.

| | $a'$ (Å) | $c$ (Å) | Total energy (eV) | Total energy (eV/f.u.) |
|---|---|---|---|---|
| Pristine $NaCrS_2$ | 7.25516 | 19.46446 | -249.85185 | -20.82099 |
| $Na_{0.75}CrS_2$ derived from Supplementary Fig. 13(b) | | | | |
| $Na_{0.75}CrS_2$ without $Cr/V^{/}_{Na}$ antisite | 7.09578 | 20.08543 | -238.33256 | -19.86105 |
| $Na_{0.75}CrS_2$ with 1/12 $Cr/V^{/}_{Na}$ antisite | 7.22277 | 19.55311 | -237.31359 | -19.77613 |
| $Na_{0.75}CrS_2$ with 1/6 $Cr/V^{/}_{Na}$ antisite | 7.19941 | 19.34232 | -236.31494 | -19.69291 |
| $Na_{0.5}CrS_2$ derived from Supplementary Fig. 13(d) | | | | |
| $Na_{0.5}CrS_2$ without antisite | 6.970676155 | 20.546766138 | -226.21639 | -18.85137 |
| $Na_{0.5}CrS_2$ with 1/6 $Cr/V^{/}_{Na}$ antisite | 7.19941 | 19.34232 | -226.00359 | -18.83363 |
| $Na_{0.5}CrS_2$ derived from Supplementary Fig. 17(a) | | | | |
| $Na_{0.5}CrS_2$ without antisite (Diffusion coordinate 0 in Supplementary Fig. 17(b)) | 7.03255 | 20.38225 | -225.89586 | -18.82466 |
| $Na_{0.5}CrS_2$ with 1/12 $Cr/V^{/}_{Na}$ antisite (Diffusion coordinate 6 in Supplementary Fig. 17(b)) | 7.10596 | 19.36157 | -225.99127 | -18.83261 |

| | | | | |
|---|---|---|---|---|
| $Na_{0.5}CrS_2$ with 1/12 Cr/$V^{/}_{Na}$ antisite (Diffusion coordinate 12 in Supplementary Fig. 17(b)) | 7.12694 | 19.45797 | -226.25580 | -18.85465 |
| $Na_{0.5}CrS_2$ with 1/12 Cr/$V^{/}_{Na}$ antisite (Diffusion coordinate 18 in Supplementary Fig. 17(b)) | 7.09847 | 19.49107 | -226.42326 | -18.86861 |

**Supplementary Table 5.** Cr-S, Na-S and S-S distances related to all S ions in $Na_{0.5}CrS_2$ with 1/6 Cr/$V'_{Na}$ antisite. Two pairs of dimers are $(S4\text{-}S22)^{2-}$ and $(S8\text{-}S14)^{2-}$, two dangling S ions are $(S10)^{-}$ and $(S20)^{-}$, and others are general $S^{2-}$. Note that the distances between Cr (Na)-S or S-S are within 3 Å. Labels of Cr and S are the same as those in Fig. 5(d-e) and Supplementary Fig. 17a.

| S | $d_{Cr\text{-}S}$ (Å) | $d_{Na\text{-}S}$ (Å) | $d_{S\text{-}S}$ (Å) |
|---|---|---|---|
| S1 | 2.460, 2.445, 2.422 | 2.992, 2.909 | |
| S2 | 2.483, 2.479, 2.506 | 2.687, 2.686 | |
| S3 | 2.447, 2.468, 2.450 | 2.859, 2.850 | |
| S4 | 2.435, 2.475 | 2.786 | 2.083 |
| S5 | 2.424, 2.427, 2.426 | 2.935 | |
| S6 | 2.446, 2.450, 2.466 | 2.840, 2.855 | |
| S7 | 2.460, 2.475, 2.454 | 2.952, 2.887 | |
| S8 | 2.470, 2.438 | 2.783 | 2.083 |
| S9 | 2.538, 2.460, 2.514, 2.468 | 2.785 | |
| S10 | 2.379, 2.379 | 2.884, 2.900 | |
| S11 | 2.412, 2.433, 2.444 | 2.935 | |
| S12 | 2.538, 2.462, 2.469, 2.514 | 2.780 | |
| S13 | 2.426, 2.423, 2.425 | 2.951 | |
| S14 | 2.433, 2.474 | 2.779 | 2.083 |
| S15 | 2.561, 2.470, 2.470, 2.500 | 2.780 | |
| S16 | 2.484, 2.481, 2.508 | 2.683, 2.681 | |
| S17 | 2.459, 2.442, 2.423 | 2.912 | |
| S18 | 2.503, 2.466, 2.557, 2.470 | 2.778 | |
| S19 | 2.413, 2.431, 2.443 | 2.925 | |
| S20 | 2.378, 2.379 | 2.897, 2.904 | |
| S21 | 2.470, 2.619, 2.469, 2.613 | 2.758 | |
| S22 | 2.438, 2.471 | 2.785 | 2.083 |
| S23 | 2.456, 2.459, 2.476 | 2.970, 2.886 | |
| S24 | 2.468, 2.468, 2.619, 2.612 | 2.763, 2.761 | |

## Supplementary Notes

**Supplementary Note 1.** Fig. 1b shows a typical high-angle annular-dark-field (HAADF) image of the atomic-level structure of pristine $NaCrS_2$ sample viewed along the [100] crystallographic direction. The atomic structure of pristine is overlaid for comparison. This direction is usually selected because the beam is parallel to the Cr, S, Na ions which line in single atomic columns, respectively, thus facilitating the convenient interpretation of contrast of the HAADF-STEM images. It is noteworthy that the contrast of the HAADF-STEM image varies with atomic number according to a $Z^{1.7}$ dependency in contrast to the annular-dark-field (ABF) technique showing a $Z^{1/3}$ relationship[1-2]. Because the HAADF technique is more sensitive to the heavy atom, Cr ions are clearly seen from the HAADF images. In Supplementary Fig. 1a, two lines are drawn in the surface (line 1) and bulk regions (line 2) of sample. The corresponding line contrast profiles from the HAADF-STEM image are shown in Supplementary Fig. 1b and c. This STEM-HAADF image is consistent with the crystal structure shown in insight of Supplementary Fig. 1a. From the STEM images and line profiles in Supplementary Fig. 1a-b, it is confirmed that the atomic arrangement of the surface structure is identical to that of the bulk, indicating a homogeneous crystal structure from the bulk to the surface region of the pristine $NaCrS_2$ sample. Furthermore, as shown in supplementary Fig. 1b, the intensity ratio between Na and Cr column is ~14.59%.

**Supplementary Note 2.** As shown in Supplementary Fig. 6, the structure evolution and lattice parameter change of $NaCrS_2$ electrode during the 3$^{rd}$ cycle are almost consistent with those during the 1$^{st}$ cycle. It can be seen that the (003) peak also keeps unchanged and (001) peak shows small shift during charge/discharge process.

**Supplementary Note 3.** In order to compare the structure evolution of $NaCrS_2$ electrode in different cells, the *in situ* XRD of $NaCrS_2$ electrode during the first charging process in Li cell was investigated and shown in Supplementary Fig. 7. As shown in XRD pattern above, the (003) diffraction peak almost keeps unchanged, while the (110) peak gradually moves towards higher $2\theta$ angle with the extraction of

Na from $NaCrS_2$. Obviously, these results are similar with the lattice change of $NaCrS_2$ electrode in Na cell.

**Supplementary Note 4.** Cluster expansion is one of the approximate methods to calculate total energy of crystal system with a mass of atoms, which was proposed by Mayer at the beginning of 1940s and employed to express the partition function as a series of expansion in powers of density[3]. In the cluster expansion, the system energy is defined by specifying the energy associated with any atomic arrangement on a given lattice[4]. The general formalism of cluster expansion parameterizes the energy as follows[5-6]:

$$E(\sigma) = \sum_{\alpha} m_{\alpha} J_{\alpha} \left\langle \prod_{p \in \alpha'} \sigma_p \right\rangle \tag{1}$$

where $\alpha$ denotes a cluster (a set of sites $p$), while $\alpha'$ is equivalent cluster of $\alpha$ by symmetry, The coefficients $J_{\alpha}$ is called the effective cluster interaction (ECI). The multiplicity $m_{\alpha}$ indicates the number of clusters that are equivalent by symmetry to $\alpha$, $\sigma_p$ is the atoms arrangement variable, $E(\sigma)$ the energy of the system with configuration $\sigma$.

Take the case of binary system (concentration of component $X$ is variable), cluster expansion in Eq. (1) could be refined as Eq. (2), in which $J$ denotes ECI, $\sigma$ structure fundamental descriptor of configuration (represented by state of component $X$, e.g. 1, if atom $X$ does occupy certain site and 0 does not, or ±1 for spin state of atom $X$). $i$, $j$, and $k$ are used to distinguish the identical site[7].

$$E(\sigma) = J_{null} + \sum_{i} J_i \sigma_i + \sum_{ij} J_{i,j} \sigma_i \sigma_j + \sum_{ijk} J_{i,j,k} \sigma_i \sigma_j \sigma_k + \cdots \tag{2}$$

where $J_{null}$, $\sum_{i} J_i \sigma_i$, $\sum_{ij} J_{i,j} \sigma_i \sigma_j$ and $\sum_{ijk} J_{i,j,k} \sigma_i \sigma_j \sigma_k$ denote the energies of empty cluster, point cluster, pair cluster and triplet cluster, respectively. If the cluster scale is accordingly increased, the obtained energy is more close to first-principles-calculated value.

Supplementary Fig. 9 shows the clusters in cluster expansion, which include point, pair and triplet clusters. The empty cluster is also included in cluster expansion

but not shown in Supplementary Fig. 9. The yellow-filled circles correspond to sites in top layer. The blue-filled circles correspond to sites in middle layer. And the purple-filled circles correspond to sites in bottom layer. The circles with character c correspond to sites in conventional cell.

a. (The calculation processes of total energies by equation (2) are detailed below: Calculate the total energies of several configurations.
b. Obtain ECI values by fitted the total energies of configurations)
c. Calculate the total energy of any selected configuration by ECI values in step b.

The ground state at certain concentration is obtained by cluster expansion method: (The ground state is the configuration with lowest formation energy at the concentration, which can be considered as most stable configuration at the concentration)

a. Determine a new configuration;
b. Calculate the cluster state of the new configuration. The cluster state presents proportional relationship with each atom which indicates the atom does (does not) occupy certain site or occupies certain site with certain spin direction.
c. Calculate the total energy of new configuration by equation (2);
d. Calculate the formation energy of new configuration;
e. As for a certain concentration, select the configuration with lowest formation energy from the configurations.

The above processes would be repetitive execution till the ground state is correctly reproduced and no new ground state is predicted at the concentration.

**Supplementary Note 5.** We explored the relative stability of $Na_{1-x}CrS_2$ with different Na concentrations ranging from $x$=0 to $x$=0.5. We considered a total of 42 configurations with many possible Na-vacancy arrangements, and calculated their corresponding formation energy per formula unit ($E_{form}(x)$, eV/f.u.) as:

$$E_{form}(x) = E[Na_{1-x}CrS_2]-xE[CrS_2]-(1-x)E[NaCrS_2] \quad (3)$$

where $E[Na_{1-x}CrS_2]$ is the total energy of an intermediate phase with a given Na composition ($x$), $E[CrS_2]$ is the total energy of the fully disodiated phase, and $E[NaCrS_2]$ is the total energy of the fully sodiated phase.

Supplementary Fig. 12a shows all calculated formation energy per formula unit as a function of Na content, from which we have built up the corresponding convex hull (convex envelope containing all the points in the plot). The convex hull connects the most stable structures such as the inflexion points by straight lines along all the composition range. According to the convex hull, Na insertion/extraction into/from $NaCrS_2$ is predicted to occur through four successive first-order transitions: between $NaCrS_2$ and $Na_{0.3333}CrS_2$, between $Na_{0.3333}CrS_2$ and $Na_{0.5}CrS_2$, between $Na_{0.5}CrS_2$ and $CrS_2$. These results are in agreement with experimental one of forming a solid solution one[8], and are similar to that of O3-$LiCoO_2$[9]. The lowest-energy configurations found at $x$=0, 1/4, 1/3, 1/2 and 1.0 are displayed in Supplementary Fig. 13.

**Supplementary Note 6.** Supplementary Fig. 13 shows the ground-state structures of different Na-vacancy concentrations for $Na_{1-x}CrS_2$ ($0 \le x \le 1$), in which (a), (b), (d) and (e) are based on 2×2×1 $NaCrS_2$ conventional cell whereas (c) √3×√3×1 $NaCrS_2$ conventional cell. It is obvious that solid-solution reaction takes place upon desodiation process. The formation energies and the corresponding coordinates of Na-vacancy in ground-state are listed in Supplementary Table 3.

**Supplementary Note 7.** As shown in Supplementary Fig. 14, we calculate the total energies of 2×2×1 $Na_{1-x}CrS_2$ ($x$=0, 1/12, 1/6, 1/4, 1/3, 5/12, 1/2) conventional cells with 1/12 $Cr/V^{/}_{Na}$ antisite relative to the corresponding those without antisite, $\Delta E$, and their lattice parameters $a'$ and $c$. It is clearly seen from Supplementary Fig. 14a that with the increase of deintercalated sodium concentration, $\Delta E$ drops from 1eV at $x$=1/12 to 0.3967eV at $x$=5/12 and 0.35179eV at $x$=1/2. This reveals that with the deintercalation of Na, the migration of Cr to Na vacancy is thermodynamically allowable. Furthermore, as compared with a sharp increase of lattice parameter $c$ of $Na_{1-x}CrS_2$ without antisite (from 19.464 Å at $x$=0 to 20.622 Å at $x$=1/2), the almost

constant lattice parameter $c$ of $Na_{1-x}CrS_2$ with 1/12 $Cr/V^{/}_{Na}$ antisite is obtained (See Supplementary Fig. 14b), which is in good agreement with the experimental result in Fig. 2c. Namely, it is the occurrence of $Cr/V^{/}_{Na}$ antisite that results in the unchanged lattice $c$. In addition, we consider the cases of $Na_{1-x}CrS_2$ with 1/6 $Cr/V^{/}_{Na}$ antisite ($x$=1/12, 1/2), and find that $\Delta E$ at $x$=1/12 reaches 3.24eV, although $\Delta E$ at $x$=1/2 is only 0.176 eV and the lattice parameter $c$ of 19.342 Å is very close to that of pristine $NaCrS_2$. Note that with the increase of deintercalated sodium concentration, there is a tendency that the lattice parameter $a$ shortens gradually, which is in agreement with the experiment result in Fig. 2b.

**Supplementary Note 8.** The redox potential for the sodium removal in $Na_xCrS_2$, *i.e.*, the average potential, can be approximately calculated via the following equation:

$$V_{ave} = -[E(NaCrS_2)-E(Na_{1-x}CrS_2)- x\,E(Na)]/xe \qquad (4)$$

where $E(NaCrS_2)$ and $E(Na_{1-x}CrS_2)$ are the total energies before and after the $x$ sodium ions extraction from the $NaCrS_2$ compound per formula unit, and $e$ is the elementary charge. The average potential of $Na_{1-x}CrS_2$ with $Cr/V^{/}_{Na}$ (Na vacancy: $V^{/}_{Na}$ in Kröger-Vink notation) antisite is calculated by total energy of $Na_{1-x}CrS_2$ with $Cr/V^{/}_{Na}$ antisite (1/12 and 1/6 $Cr/V^{/}_{Na}$ antisites in $Na_{0.75}CrS_2$ and $Na_{0.5}CrS_2$, respectively). The entropy, volume change, and temperature effects are ignored. We choose energetically the most favorable configuration.

The GITT profiles of $Na_{1-x}CrS_2$ (we define the $Na_{1-x}CrS_2$ electrode charged to 3.0 V with $x$=0.5 as fully charged sample in the first charge process according to the capacity) and the computed average voltage along the minimum energy path of formation energies are shown in Supplementary Fig. 15. When $x$=0.25, 0.33, 0.5, the calculated average voltage are 2.610, 2.652 and 2.680 V for $Na_{1-x}CrS_2$ without antisite. While the average voltage are 2.910 and 2.962 V for $Na_{1-x}CrS_2$ with $Cr/V^{/}_{Na}$ antisite when $x$=0.25, 0.5. These values agree well with the first experimental charging profile.

**Supplementary Note 9.** The possible environments (a) and corresponding energy barriers (b) for an *o-t-o* Na hopping in $Na_{0.75}CrS_2$ and $Na_{0.5}CrS_2$ are illustrated in Supplementary Fig. 16. In layered $NaCrS_2$, both Na and Cr occupy in octahedral sites, and Na diffusion proceeds by hopping from one octahedral site to another octahedral site via an intermediate tetrahedral site (*o-t-o* diffusion), which is an activated state in Na diffusion. This is similar to the cases of most of disordered rocksalt[10-12].

The Na hopping barrier in $Na_{0.75}CrS_2$ without $Cr/V^{/}_{Na}$ antisite is about 0.51 eV, whereas with the further Na deintercalation, the corresponding value is reduced to 0.37 eV for $Na_{0.667}CrS_2$ without antisite. (See ② in Supplementary Fig. 16). In order to examine the effect of $Cr/V^{/}_{Na}$ antisite on Na diffusion, we calculate the Na diffusion barriers in $Na_{0.5}CrS_2$ and $Na_{0.417}CrS_2$ with 1/6 $Cr/V^{/}_{Na}$ antisite. As shown in ③ and ④ in Supplementary Fig. 16a, there is each possible Na diffusion *o-t-o* pathway for two cases with 1/6 $Cr/V^{/}_{Na}$ antisite. The corresponding migration barriers in Supplementary Fig. 16b are 0.33eV and 0.27eV, respectively, which are close to the typical 1-TM barrier values (~0.3eV) in layered oxides[11]. As compared with ① and ②, Na diffusions in $Na_{0.5}CrS_2$ with $Cr/V^{/}_{Na}$ antisite exhibit the lower energy barriers. This can be explained from: (i) the reduction of the tetrahedron height due to the $Cr/V^{/}_{Na}$ antisite, which is similar to that in the disordered rocksalt; (ii) the strong electrostatic repulsion on an activated Na and Cr ions. On the other hand, this also reveals that the occurrence of $Cr/V^{/}_{Na}$ antisite induced due to the stepwise Na deintercalation does not hinder the Na diffusion, which is similar to that of cation-disordered oxides for rechargeable lithium batteries[10].

**Supplementary Note 10.** Supplementary Fig.17 shows the migration process of Cr in $Na_{0.5}CrS_2$: (a) the migration path and (b) the corresponding energy barrier profile, which involves three substeps and is essentially a formation process of $Cr/V^{/}_{Na}$ antisite. During the substep1, via the cross-layer migration of 3.548 Å, Cr (Cr3) travels from its lattice site A to the Na vacancy at site B, which is accompanied by an energy barrier of 0.83 eV. It is worth mentioning that although there is no the $Cr/V^{/}_{Na}$ antisite for the initial state (diffusion coordinate 0) during the substep 1, it is a little different from the ground state of $Na_{0.5}CrS_2$ without the $Cr/V^{/}_{Na}$ antisite (See

Supplementary Fig. 17a and Supplementary Fig. 13d for 2×2×1 $Na_{0.5}CrS_2$ conventional cells). For the former, the $Na^+$ number of the middle Na layer is less than those of the two-sided ones so as to ensure the enough Na vacancies for Cr migration, whereas for the latter three Na layers have the same $Na^+$ numbers. Seeing from the energetics, the total energy of the former is higher than that of the latter by 0.0801eV/f.u., which signifies that it is entirely possible for both states to be existent during the electrochemical discharge/discharge process. The next migration occurs in the Na intra-layer, as shown during the substep 2, Cr (Cr3) travels from the Na vacancy at site B to another Na vacancy at site C by the distance of 3.446 Å and with the energy barrier of 0.85 eV. Finally, during the substep 3, via the divacancy mechanism similar to Li hopping in rocksalt-like Li-TM oxides[10], Na (Na5) migrates from the lattice site D to the vacancy at site B (regenerated due to the migration of Cr (Cr3) during the substep 2) by the travel distance of 3.629 Å and with the energy barrier of about 0.47 eV.

## Supplementary References


1. Findlay, S. D. *et al.* Atomic resolution imaging of light elements using scanning transmission electron microscopy. *Appl. Phys. Lett.* **95**, 191913 (2009).
2. Findlay, S. D. *et al.* Dynamics of annular bright field imaging in scanning transmission electron microscopy. *Ultramicroscopy* **110**, 903–923 (2010).
3. Mayer, J. & Montroll, E. Molecular distributions. *J. Chem. Phys.* **9**, 2–16 (1941).
4. Van de Walle, A. & Asta, M. Self-driven lattice-model Monte Carlo simulations of alloy thermodynamic properties and phase diagrams. *Modelling Simul. Mater. Sci. Eng.* **10**, 521–538 (2002).
5. Garbulsky, G. & Ceder, G. Linear-programming method for obtaining effective cluster interactions in alloys from total-energy calculations: application to the fcc Pd-V system. *Phys. Rev. B* **51**, 67–72 (1995).
6. Van de Walle, A.， Asta, M. & Ceder, G. The alloy theoretic automated toolkit: A user guide. *Calphad* **26**, 539–553 (2002).
7. Meng, Y. & Arroyo-de Dompablo, M. First principles computational materials design for energy storage materials in lithium ion batteries. *Energy Environ. Sci.* **2**, 589–609 (2009).
8. Yue, J. L. *et al.* Discrete Li-occupation versus pseudo-continuous Na-occupation and their relationship with structural change behaviors in $Fe_2(MoO_4)_3$. *Sci. Rep.* **5**, 8810 (2015).
9. Van der Ven, A., Aydinol, M. K. & Ceder, G. First-principles investigation of phase stability in $Li_xCoO_2$. *Phys. Rev. B* **58**, 2975–2987 (1998).
10. Lee, J. *et al.* Unlocking the potential of cation-disordered oxides for rechargeable lithium batteries. *Science* **343**, 519–522 (2014).
11. Kang, K. & Ceder, G. Factors that affect Li mobility in layered lithium transition metal oxides. *Phys. Rev. B* **74**, 094105 (2006).
12. Van der Ven, A. & Ceder, G. Lithium diffusion in layered $Li_xCoO_2$. *Electrochem. Solid State Lett.* 3, 301–304 (2000).
